# Effects of glutamine, glutamate, and aspartate on intestinal barrier integrity and amino acid pool of the small intestine in piglets with normal or low energy diet

**DOI:** 10.3389/fvets.2023.1202369

**Published:** 2023-07-27

**Authors:** Yuankun Deng, Hao Cheng, Junyao Li, Hui Han, Ming Qi, Nan Wang, Bi'e Tan, Jianjun Li, Jing Wang

**Affiliations:** ^1^College of Animal Science and Technology, Hunan Agricultural University, Changsha, Hunan, China; ^2^Laboratory of Animal Nutritional Physiology and Metabolic Process, Key Laboratory of Agroecological Processes in Subtropical Region, National Engineering Laboratory for Pollution Control and Waste Utilization in Livestock and Poultry Production, Institute of Subtropical Agriculture, Chinese Academy of Sciences, Changsha, Hunan, China

**Keywords:** glutamine, glutamate, aspartate, amino acid pool, intestinal barrier

## Abstract

Aspartate (asp), glutamate (glu), and glutamine (gln) are the major energy fuels for the small intestine, and it had been indicated in our previous study that the mix of these three amino acid supplementations could maintain intestinal energy homeostasis. This study aimed to further investigate whether the treatment of gln, glu, and asp in low energy diet affects the intestinal barrier integrity and amino acid pool in weaning piglets. A total of 198 weaned piglets were assigned to 3 treatments: control (basal diet + 1.59% L-Ala); T1 (basal diet + 1% L-Gln + 0.5% L-Glu + 0.1% L-Asp); and T2 (low energy diet + 1% L-Gln + 0.5% L-Glu + 0.1% L-Asp). The blood, jejunum, and ileum were obtained on day 5 or on day 21 post-weaning, respectively. Our results showed that T1 and T2 treatments increased the abundances of occludin, claudin-1, and claudin-3 in the small intestine while decreasing the serum diamine oxidase (DAO) and D-lactate levels in weaning piglets. Meanwhile, T1 and T2 treatments significantly increased the positive rate of proliferating cell nuclear antigen (PCNA) of the small intestine, promoting intestinal cell proliferation. We also found that supplementation with glu, gln, and asp improved the serum amino acid pool and promoted ileal amino acid transporter gene expression of *slc3a2, slc6a14*, and *slc7a11* in weaned piglets. Additionally, on day 21 post-weaning, T1 and T2 treatments stimulated the phosphorylation of the mTOR-S6K1-4EBP1 signaling pathway in the small intestine, which may implicate the enhanced protein synthesis rate. In summary, dietary supplementation of gln, glu, and asp was beneficial to the intestinal barrier function and amino acid pool regulation, while the benefits of gln, glu, and asp treatment might be diminished by the low-energy diet. The results demonstrated that the supplementation of gln, glu, and asp under low energy levels was preferentially supplied as the energy fuel to restore the gut barrier function in piglets on day 5 post-weaning. With the increase in age and intestinal maturation (on day 21 post-weaning), gln, glu, and asp supplementation could also show an effect on the regulation of the amino acid pool and protein synthesis.

## 1. Introduction

The small intestine, which plays key roles in nutrient digestion and absorption, acts as an innate barrier against luminal pathogens and physiological stress (1). Intestinal dysplasia is one of the most important causes of morbidity and mortality in piglets during weaning (2). First, environmental changes may disturb the secretion of hormones, such as cortisol, which may occur when piglets are removed from their dam, transported, and relocated (3–5). Second, food changes may cause damage to the intestinal mucosal barrier and decrease the activities of brush border enzymes (6, 7). After intestinal injury, weaned piglets mainly rely on the continuous proliferation, differentiation, and migration of intestinal epithelial cells to rebuild the integrity of the intestinal epithelium and maintain the function of the intestinal mucosal barrier, which requires a number of ATPs to support it (8, 9). However, the low feed intake and the immature intestines of new-weaning piglets determine that they are unable to fully digest and absorb nutrients, which, in turn, leads to insufficient energy supply for piglets, especially, for the intestine.

Gln, glu, and asp are the major energy sources of the small intestine epithelial cells (10). Our previous study has shown that dietary pattern supplemented with asp, glu, and gln under normal or low energy status can maintain the energy homeostasis of small intestinal mucosa in piglets by either replenishing the Krebs cycle or downregulating the AMPK (11). The results of our previous study indicated that the regulation of asp, glu, and gln on the rebuilding of intestinal mucosa in piglets after weaning might change according to piglets' energy status.

Meanwhile, these amino acids also play key roles in regulating amino acid pool and protein synthesis, which are essential for gut epithelial proliferation, differentiation, and mucosa remodeling (10, 12–14). mTOR signaling pathway is the main amino acid target, which can increase intestinal protein synthesis and epithelial repair. mTOR phosphorylates its downstream targets, ribosomal protein S6 kinase (S6K) and eukaryotic initiation factor 4E-binding protein 1 (4E-BP1), thereby promoting intestinal mRNA translation, protein synthesis, and cell growth. The tight junction proteins also play key roles in restoring the epithelial barrier function (15). The dysregulation of tight junction might disrupt the intestinal barrier homeostasis (16).

Based on our previous study (11), in this study, we aim to further evaluate the effect of asp, glu, and gln supplementation on the intestinal barrier integrity and amino acid pool in the small intestine of weaning piglets on day 5 and day 21 post-weaning under different energy statuses.

## 2. Materials and methods

### 2.1. Ethical statement

All experimental and sample collection procedures were carried out as per the Chinese guidelines for animal welfare and approved by the Institutional Animal Care and Use Committee of Hunan Agricultural University.

### 2.2. Animals, diets, and management

A total of 198 piglets (Duroc × Landrace × Large Yorkshire) weaned at 21 days of age were assigned to 18 pens based on their body weight (BW). There were 11 piglets per pen and 6 pens per treatment. The treatments include: (i) T0 (basal diet +1.59% L-Ala; iso-nitrogenous control); (ii) T1 (basal diet + 0.1% L-Asp + 1% L-Gln + 0.5% L-Glu); and (iii) T2 (low energy diet + 0.1% L-Asp + 1% L-Gln + 0.5% L-Glu). The basal diets were formulated to meet the nutrient requirements recommended by the National Research Council (17) (Table 1). A low energy level diet removed soybean oil, glucose, and sucrose, and the doses of asp, glu, and gln were based on previous studies (11). All the piglets were housed in an environmentally well-controlled nursery facility with slatted plastic flooring and a mechanical ventilation system and had free access to drink water.

**Table 1:** Ingredient composition of diets^a, b^.

**Item**	**T0**	**T1**	**T2**
**Ingredient (%)**			
Corn	23.93	24.00	24.40
Extruded corn	35.00	35.00	35.00
Soybean	8.00	8.00	11.80
Fermented soybean	9.00	9.00	4.00
Extruded soybean	0.00	0.00	2.80
Whey powder	6.00	6.00	6.00
Fish meal	4.00	4.00	4.00
Plasma protein powder	2.00	2.00	2.00
Soybean oil	1.00	1.00	0.00
Glucose	3.00	3.00	0.00
Sucrose	2.00	2.00	2.00
L-lysine	0.40	0.40	0.40
DL-methionine	0.11	0.11	0.11
L-threonine	0.12	0.12	0.12
L-alanine	1.59	0.00	0.00
L-glutamine	0.00	1.00	1.00
L-glutamate	0.00	0.50	0.50
L-aspartate	0.00	0.10	0.10
Carrier	0.90	0.82	0.82
Organic acid calcium	0.60	0.60	0.60
CaHPO_4_	1.00	1.00	1.00
Choline chloride (50 %)	0.01	0.01	0.01
Antioxidant	0.05	0.05	0.05
Mineral premix^a^	0.15	0.15	0.15
Vitamin premix^b^	0.04	0.04	0.04
ZnO	0.40	0.40	0.40
Acidifier	0.70	0.70	0.70
Total	100.00	100.00	100.00
**Nutrient composition** ^*^			
Digestible energy (kCal/kg)	3445.60	3444.56	3227.00
Crude protein	19.57	19.53	19.53
Calcium	0.47	0.47	0.47
Total phosphorus	0.40	0.40	0.40
Total lysine	1.14	1.14	1.11

^a^Miner premix provided for 1 kg completed diet: Zn (ZnO), 50 mg; Cu (CuSO_4_), 20 mg; Mn (MnO), 55 mg; Fe (FeSO_4_), 100 mg; I (KI), 1 mg; Co (CoSO4), 2 mg; Se (Na2SeO3), 0.3 mg. ^b^Vitamin premix provided for 1 kg completed diet: vitamin A, 8,255 IU; vitamin D3, 2,000 IU; vitamin E, 40 IU; vitamin B1, 2 mg; vitamin B2, 4 mg; pantothenic acid, 15 mg; vitamin B6, 10 mg; vitamin B12, 0.05 mg; nicotinic acid, 30 mg; folic acid, 2 mg; vitamin K3, 1.5 mg; biotin, 0.2 mg; choline chloride, 800 mg; and vitamin C, 100 mg. ^*^Calculated.

### 2.3. Sample collection

On day 5 and day 21 after weaning, blood samples (5 ml) were collected in heparinized tubes. Serum was, then, centrifuged at 3,000 × *g* at 4°C for 10 min and stored in 1.5 ml centrifugal tubes at −80°C for further analysis. Subsequently, one piglet per replicate was randomly selected to be euthanized and necropsied after blood sampling. Jejunal and ileal mucosa were collected.

### 2.4. Immunohistochemical staining

Jejunal and ileal intestinal tissue segments were collected, fixed for 24 h in 10% formalin, dehydrated according to the standard protocol, and cut into paraffin sections. After dewaxing, hydration, and antigen retrieval, the tissue sections were incubated for 10 min in 3% H_2_O_2_ and washed with 0.01 mol/L PBS. The sections were then incubated with PCNA polyclonal antibody (1:200, Cell Signaling Technology), claudin 1 polyclonal antibody (1:200, Cell Signaling Technology), claudin 3 polyclonal antibody (1:200, Cell Signaling Technology), and occludin polyclonal antibody (1:200, Cell Signaling Technology), followed by incubation with the corresponding horseradish peroxidase-labeled secondary antibody. The proteins were then developed in 3,3′-diaminobenzidine for coloration and assessment. The stained sections were acquired using a computer-assisted microscope (Olympus, Tokyo, Japan) and scored independently using Image-J2 software. The PCNA labeling index was expressed as the ratio of cells that were positively stained for PCNA in all epithelial cells, and the abundances of tight junction proteins were expressed as the average optical density (the ratio of integral optical density to the area of tissue) in at least 6 areas that were randomly selected for counting at 400-fold magnification.

### 2.5. Serum DAO and d-lactate level analysis

The serum DAO (Catalog # A088) and D-lactate (Catalog # A019) contents were measured using commercial kits purchased from Nanjing Jiancheng Bioengineering Institute (Nanjing, China).

### 2.6. Serum of amino acid

The free amino acids of serum were analyzed using a high-performance liquid chromatography system (HPLC, LA8080, Japan), according to the manufacturer's instructions. The preprocessing of the samples was conducted following the description. First of all, 1 ml of serum samples were homogenized with 1 ml of 0.01 M hydrochloric acid and then extracted using ultrasonic extraction for 30 min. Next, 1 ml of supernatants was mixed with 0.8% sulfosalicylic acid solution. After being incubated at 4°C for 15 min, the mixtures were then centrifuged at 10,000 rpm for 20 min and filtered using a 0.22-μm filter membrane before being analyzed using the HPLC system.

### 2.7. Na^+^-k^+^ ATPase activity in the ileum and jejunum

The jejunal and ileal mucosa tissues of Na^+^-K^+^ ATPase (Catalog # A070) activity were measured using a commercial kit purchased from Nanjing Jiancheng Bioengineering Institute (Nanjing, China).

### 2.8. Real-time quantitative PCR

The expressions of *SLC1A1, SLC1A5, SLC3A1, SLC3A2, SLC36A1, SLC7A2, SLC38A2, SLC6A19, SLC6A20, SLC7A1, SLC7A7, SLC7A9, SLC16A10, SLC38A9, SLC6A14*, and *SLC7A11* in mRNA in the jejunal and ileal mucosa were determined using real-time quantitative reverse transcriptase PCR (real-time qPCR), as described previously (11). Primers were designed with Primer 5.0 (PREMIER Biosoft International, Palo Alto, CA) according to the gene sequence of the pig to produce an amplification product (Table 2 primer sequences). The comparative threshold cycle (Ct) value method (2^−ΔΔ*Ct*^) was employed to quantitate expression levels for target genes relative to those for the β-Actin. Data were expressed as the relative values to those of control piglets at day 5 post-weaning.

**Table 2:** Primers used for real-time quantitative reverse transcription-PCR^a^.

**Gene**	**Accession no**.	**Primers**
*β-Actin*	XM_003124280.3	F: GGATGCAGAAGGAGATCACG
		R: ATCTGCTGGAAGGTGGACAG
*SLC1A1*	NM_001164649.1	F: GGCGCGGTGAGCAATCTC
		R: GAGCGCAGTGAAGGAAGGT
*SLC1A5*	XM_003127238.5	F: CTGTTCCCTCCCGGGATTC
		R: TGGAAGACAGAGCTCCAGGAA
*SLC3A1*	NM_001123042.1	F: CAGCTGAGGTAACAGAGGCTT
		R: TCCAAGTGAAAACCTCCTTTTCT
*SLC3A2*	XM_003353809.4	F: ACGCTCCGTCTTGGTCATAG
		R: ACCCTGCACACAGGTTTAGG
*SLC36A1*	XM_003134140.5	F: AGGAAAGCAGCAGCCTCG
		R: GGGTTCCGACTTGAGCTCTG
*SLC7A2*	XM_021077148.1	F: GTGCCCAGTCCGACTTCTG
		R: GAACGGCTGGAGCTTGTCAG
*SLC38A2*	XM_003126626.6	F: GCCCACTGACGCGTACTCC
		R: GCCGTCTGAAAGGAAAGGCG
*SLC6A19*	XM_003359855.4	F: TTGGCGCCTCTGCAGATAAC
		R: GGGAACCTTTGGCACCTCAG
*SLC6A20*	XM_021068639.1	F: ATCCATTTACCAGCCTCGCC
		R: GCTTCGGCCTGGGGATG
*SLC7A1*	NM_001012613.1	F: CGCCTTCGACTCTCCATTCA
		R: GAGTGGAAGCTCGGGTAACG
*SLC7A7*	XM_011772364.2	F: GCGATCCAGGTTTTTGAGGCA
		R: TAAGCAGGTTCTCACGGCAG
*SLC7A9*	NM_001110171.1	F: GAACCCAAGACCACAAATC
		R: ACCCAGTGTCGCAAGAAT
*SLC16A10*	XM_021091212.1	F: CTGCCTGTTCAGTCCGGG
		R: CTAGTGAGGCCGAGGCAAG
*SLC38A9*	XM_021077047.1	F: AGGCCGTTGTCTTTTCCTGT
		R: GAAGGCTGACACCCCTTACC
*SLC6A14*	NM_001348402.1	F: ATGGACAGGTTGAAGTGCCC
		R: CCAGTTACCACGGTCCTGAT
*SLC7A11*	XM_021101587.1	F: AGCTTAAATACCAGCCCACGA
		R: AGAAACACCTGTGTGTTCGGT

^a^SLC1A1, solute carrier family 1 member 1; SLC1A5, solute carrier family 1 member 5; SLC3A1, solute carrier family 3 member 1; SLC3A2, solute carrier family 3 member 2; SLC36A1, solute carrier family 36 member 1; SLC7A2, solute carrier family 7 member 2; SLC38A2, solute carrier family 38 member 2; SLC6A19, solute carrier family 6 member 19; SLC6A20, solute carrier family 6 member 20; SLC7A1, solute carrier family 7 member 1; SLC7A7, solute carrier family 7 member 7; SLC7A9, solute carrier family 7 member 9; SLC16A10, solute carrier family 16member 10; SLC38A9, solute carrier family 38 member 9; SLC6A14, solute carrier family 6 member 14; SLC7A11, solute carrier family 7 member 11; F, forward; R, reverse.

### 2.9. Western blot

The Western blot was used to detect liver kinase B1 (LKB1), ras homolog enriched in the brain (Rheb), tuberous sclerosis complex 2 (TSC2), raptor, mTOR, S6K1, and 4EBP1 phosphorylation and expression levels of total proteins in jejunal and ileal mucosa, respectively. The jejunal and ileal mucosa samples were homogenized, and protein concentrations were measured using the bicinchoninic acid assay method with BSA as standard (Catalog# P0010, Beyotime Institute of Biotechnology, Shanghai, China). The following first antibodies were used for protein quantification: LKB1 (1:1,000, Cell Signaling Technology); Raptor (1:1,000, Cell Signaling Technology); Rheb (1:1,000, Cell Signaling Technology); mTOR (1:1,000, Cell Signaling Technology); TSC2 (1:1,000, Cell Signaling Technology); S6K1 (1:1,000, Cell Signaling Technology); 4EBP1 (1:1,000, Cell Signaling Technology); p-mTOR (1:1,000, Cell Signaling Technology); p-TSC2 (1:1,000, Cell Signaling Technology); p-S6K1 (1:1,000, Cell Signaling Technology); and p-4EBP1 (1:1,000, Cell Signaling Technology) and p-4EBP1(1:1,000, Cell Signaling Technology).

### 2.10. Statistical analysis

**Figure 1:**
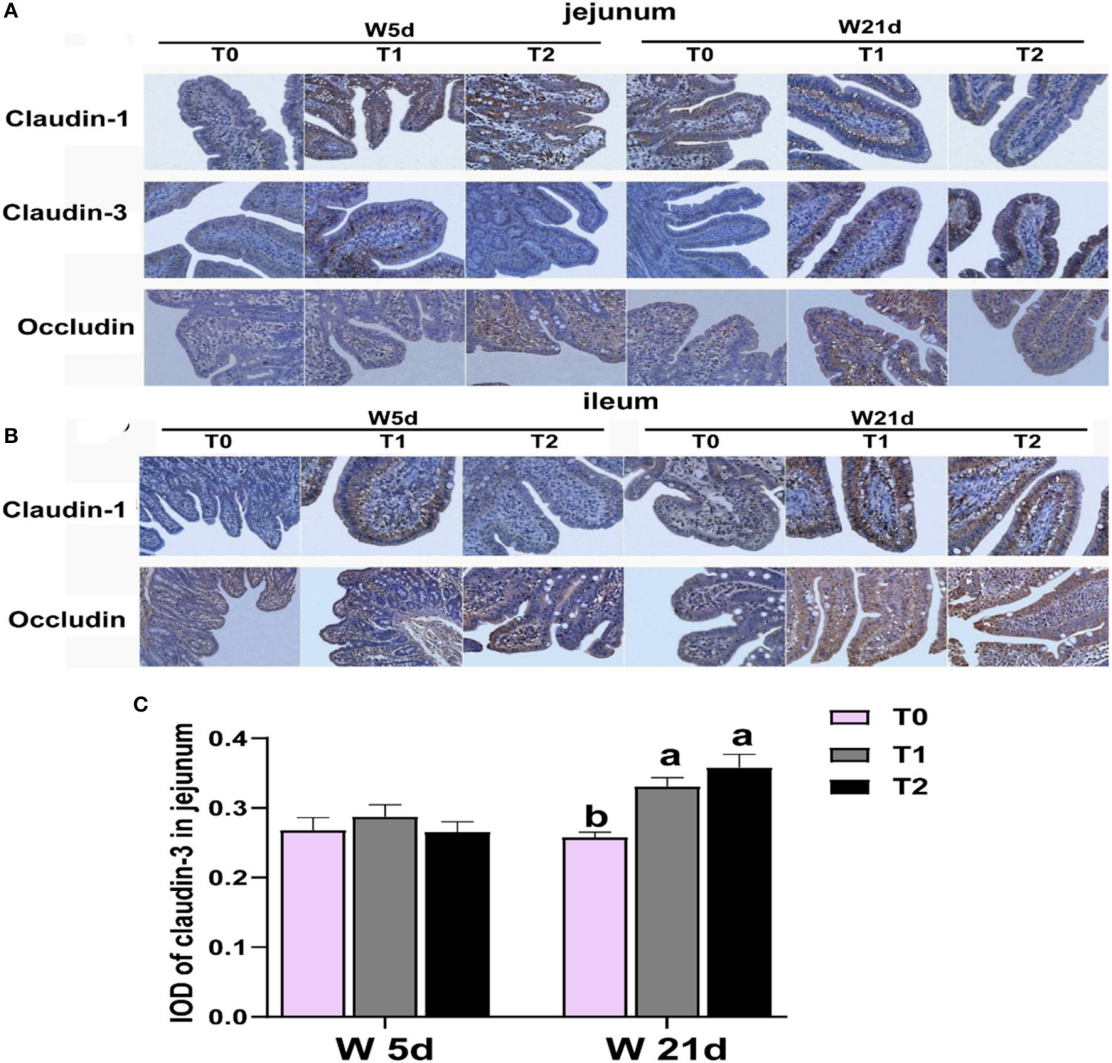
Relative abundances of Figure intestinal tight junction protein in the ileum and jejunum of weaning piglets. **(A, B)** Images of immunohistochemical staining in the small intestine of piglets. **(C)** The relative abundance of claudin 3 in the jejunum of piglets. The results are expressed as integrated optical density (IOD). ^a − c^Values with different letters within the same row are different (*P* < 0.05). 2W 5d = days 5 post-weaning piglets; W21d = days 21 post-weaning piglets. 3T0 = basal diets containing 1.59 % L-Ala (iso-nitrogen); T 1 = basal diet containing 1 % L-Gln, 0.5 % L-Glu, and 0.1 % L-Asp; T 2 = energy deficiency diet containing 1 % L-Gln, 0.5 % L-Glu, and 0.1 % L-Asp. n=6, the same as below.

Data were statistically analyzed by the procedure of general linear models using SPSS 19.0 (SPSS Inc., Chicago, IL). The results were expressed as arithmetic mean. Diet treatments, days after weaning, and the interaction between treatment and day as the main effects. The results in the tables are presented as the main effects. When no significant interaction between treatment and day was found, the differences between treatments and days were evaluated using Tukey's HSD test. When significant interaction between treatments and day was found, a simple main effect analysis would be performed, and the differences among 3 treatments across 2 levels of days post-weaning were evaluated using the Bonferroni test (shown in Figures 1, 3, 4). The developmental changes of the intestine were not the main outcomes, so the differences between day 5 and day 21 post-weaning across the three levels of treatments were not shown. Differences were declared as significant at *P* < 0.05.

## 3. Results

### 3.1. Asp, glu, and gln reduced the gut permeability of weaning piglets

First, we tested the expression of intestinal tight junction protein of weaning piglets. The main effects of the diet treatments and days after weaning on tight junction protein expression are shown in Table 3. T1 treatments increased the proteinabundance of claudin-1 in the jejunum and occludin in the ileum (*P* < 0.05). The protein expressions of claudin-3 in the jejunum were enhanced by T1 and T2 treatments as compared with the T0 treatments (*P* < 0.05). There were interactions between treatments and days on the jejunal claudin-3 abundance, which means that days after weaning influenced the efficacy of the treatments (*P* < 0.05). In Figure 1, T1 and T2 treatments significantly increased the abundance of jejunal claudin-3 on day 21 post-weaning (*P* < 0.05), but the post-weaning on day 5 was not significantly different (*P* > 0.05). Serum DAO and D-lactate are the main markers of the permeability of the intestinal mucosal barrier. T1 and T2 treatments significantly decreased the serum DAO and D-lactate levels in weaning piglets (*P* < 0.05), but the days post-weaning did not affect the efficacy of diet treatment on the proliferation of intestinal cells (*P* > 0.05) (Table 4).

**Table 3:** Relative abundances of small intestinal tight junction protein in the ileum and jejunum of weaning piglets.

**Item**	**Diet treatment**			**Day post-weaning**		**SEM**			***P*** **-value**	
	**T0**	**T1**	**T2**	**5**	**21**	**Diet**	**Day**	**Diet**	**Day**	**Diet**×**Day**
**Jejunum**										
Occludin	0.32^b^	0.38^a^	0.36^ab	0.34	0.38^*^	0.012	0.01	0.004	0.017	0.395
Claudin 1	0.34^b^	0.41^a^	0.37^b	0.35	0.40^*^	0.011	0.009	0.001	0.002	0.77
Claudin 3	0.26^b^	0.31^a^	0.31^a	0.27	0.32^*^	0.011	0.009	0.004	0.002	0.009
**ileum**										
Occludin	0.30^b^	0.34^a^	0.32^b	0.3	0.34^*^	0.007	0.006	0.001	0	0.055
Claudin 1	0.43	0.45	0.46	0.47^*^	0.42	0.014	0.012	0.523	0.005	0.191
Claudin 3	0.24	0.24	0.48	0.25	0.23	0.011	0.009	0.064	0.242	0.064

^a, b^Values with different letters within the same row are different (*P* < 0.05); ^*^means the difference was significant when compared to d 5 post-weaning (*P* < 0.05); *n* = 6. The results are presented as main effects, and the differences among treatments or days are evaluated using Tukey's HSD test.

**Table 4:** Serum DAO (U/ml) and D-lactate (mmol/μl) of level in the weaning piglets.

**Item**	**Diet treatment**			**Day post-weaning**		**SEM**			***P*** **-value**	
	**T0**	**T1**	**T2**	**5**	**21**	**Diet**	**Day**	**Diet**	**Day**	**Diet**×**Day**
DAO	44.07^a^	35.25^b	38.31^b^	41.77^*^	36.65	1.276	1.042	0	0.002	0.692
D-lactate	14.70^a^	11.22^b	12.22^b^	13.84^*^	11.58	0.695	0.568	0.004	0.009	0.426

^a, b^Values with different letters within the same row are different (*P* < 0.05); ^*^means the difference was significant when compared to d 5 post-weaning (*P* < 0.05); *n* = 6. The results are presented as main effects, and the differences among treatments or days are evaluated using Tukey's HSD test.

### 3.2. Asp, glu, and gln promote the proliferation of intestinal cells in the ileum and jejunum of piglets

**Figure 2:**
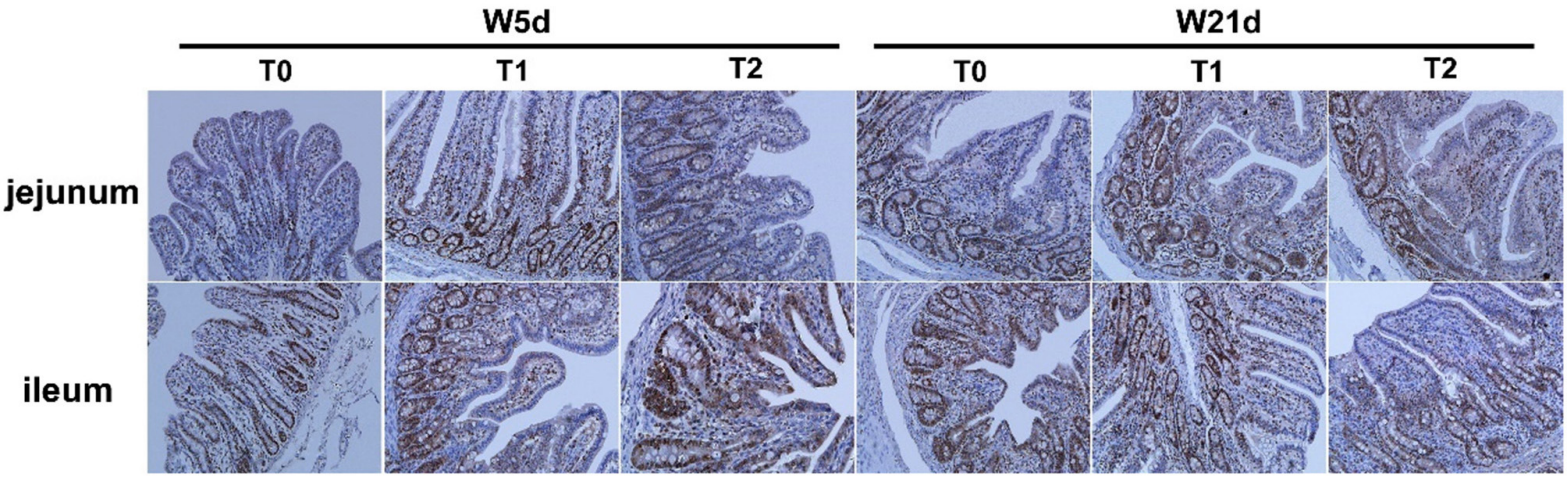
Effect of the positive rate of PCNA on the ileum and jejunum of weaning piglets. Images of immunohistochemical staining of the small intestine of piglets.

The PCNA positive rates of jejunal and ileal mucosa are presented in Table 5 and Figure 2. The T1 treatment significantly increased the positive rate of PCNA in jejunal mucosa (*P* < 0.05). Days post-weaning did not affect the efficacy of diet treatments on the intestinal PCNA positive rate (*P* > 0.05).

**Table 5:** The positive rate of proliferating cell nuclear antigen (PCNA) in the ileum and jejunum of weaning piglets.

**Item**	**Diet treatment**			**Day post-weaning**		**SEM**			***P*** **-value**	
	**T0**	**T1**	**T2**	**5**	**21**	**Diet**	**Day**	**Diet**	**Day**	**Diet**×**Day**
**Jejunum**										
PCNA	0.40^b^	0.47^a^	0.44^ab^	0.41	0.46^*^	0.014	0.012	0.003	0.001	0.718
**ileum**										
PCNA	0.38	0.44	0.43	0.42	0.41	0.017	0.008	0.069	0.829	0.967

^a, b^Values with different letters within the same row are different (*P* < 0.05); ^*^means the difference was significant when compared to d 5 post-weaning (*P* < 0.05); *n* = 6. The results are presented as main effects, and the differences among treatments or days are evaluated using Tukey's HSD test.

### 3.3. Asp, glu, and gln change the serum amino acid pool of weaning piglets

**Figure 3:**
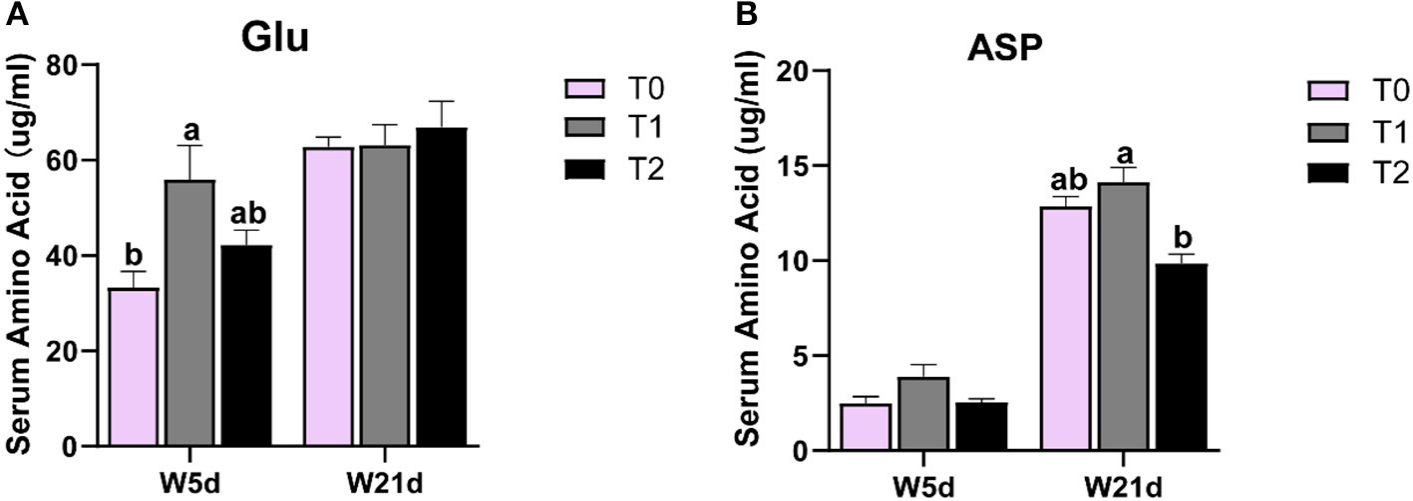
The change of asp and glu levels in the serum of weaning piglets. **(A, B)** glu and asp levels in the serum of weaning piglets on different days.

As shown in Table 6, the serum amino acids were affected by dietary gln, glu, and asp treatments, as well as days after weaning. The serum asp content in the T1 treatment was higher than that in T0 and T2 treatments (*P* < 0.05), whereas serum phe and his levels in the T1 treatment were lower than those in the T2 treatment (*P* < 0.05). Meanwhile, supplementation of gln, glu, and asp in a low-energy diet (T2) increased the lys, arg, and thr levels in serum as compared with the T0 treatment (*P* < 0.05). There were interactions between treatments and days on the serum asp and glu contents (*P* < 0.05), which means that days after weaning influenced the efficacy of the treatments. In Figure 3, the T1 treatment increased glu content on day 5 post-weaning as compared with the T0 treatment (*P* < 0.05), while T2 treatments decreased asp level on day 21 post-weaning as compared with the T1 treatments (*P* < 0.05).

**Table 6:** Serum amino acid pool in weaning piglets (ug/ml).

**Item**	**Diet treatment**			**Day post-weaning**		**SEM**			***P*** **-value**	
	**T0**	**T1**	**T2**	**5d**	**21d**	**Diet**	**Day**	**Diet**	**Day**	**Diet** × **Day**
Asp	7.69^b^	9.03^a^	6.21^ab^	2.99	12.29^*^	0.371	0.425	0	0	0.008
Ser	17.46	18.48	18.9	12.5	24.06^*^	1.385	1.161	0.752	0	0.278
Glu	48.07	59.58	54.62	43.85	64.33^*^	3.197	3.683	0.051	0	0.047
Met	6.27	7.1	7.57	6.49	7.47	0.479	0.544	0.149	0.078	0.34
Ile	15.36	15.29	17.31	16.69	15.28	1.536	1.772	0.575	0.43	0.736
Leu	21.56	22.63	25.01	22.27	23.86	1.597	1.849	0.306	0.396	0.62
Tyr	8.38	9.82	10.69	8.5	10.76^*^	0.942	1.098	0.233	0.046	0.588
Phe	18.29^b^	19.25^b^	21.56^a^	17.44	21.96^*^	0.651	0.754	0.004	0	0.425
Lys	33.45^b^	37.51^ab^	43.21^a^	32.31	43.81^*^	2.151	2.483	0.012	0	0.514
His	10.05^ab^	9.97^b^	11.44^a^	12.78^*^	8.19	0.413	0.488	0.029	0	0.159
Trp	4.98	5.45	6.19	5.58	5.51	0.465	0.534	0.182	0.886	0.729
Arg	24.55^b^	28.00^ab^	30.53^a^	21.95	33.43^*^	1.274	1.478	0.009	0	0.637
Thr	48.30^b^	54.23^ab^	59.53^a^	46.63	61.40^*^	3.073	3.554	0.049	0	0.537
Gly	76.21	70.98	71.76	48.44	97.53^*^	3.927	4.533	0.601	0	0.247
Ala	54.48	46.59	53.4	43.01	60.97^*^	4.059	4.688	0.342	0	0.169
Cys	12	12.67	13.27	14.01	11.28	1.872	2.163	0.891	0.216	0.978
Val	28.03	27.72	31.15	29.03	28.9	2.441	2.818	0.551	0.961	0.798
Pro	17.21	17.67	16.16	13.73	20.29^*^	1.708	1.961	0.812	0.002	0.999

^a, b^Values with different letters within the same row are different (*P* < 0.05); ^*^means the difference was significant when compared to d 5 post-weaning (*P* < 0.05); *n* = 6. The results are presented as main effects, and the differences among treatments or days are evaluated using Tukey's HSD test.

### 3.4. Effect of asp, glu, and gln on intestinal amino acid transporters of piglets

To determine whether the changes in the serum amino acid pool are influenced by their transferases, we examined the mRNA gene expressions of relevant amino acid transferases in jejunal and ileal mucosa. As shown in Table 7, the T1 treatment significantly increased the jejunal mucosa *SLC7A2* mRNA expression level (*P* < 0.05) but decreased the ileal mucosa *SLC1A5, SLC3A1, SLC36A1, SLC3A2, SLC6A14*, and *SLC7A11* mRNA levels as compared with the T2 treatment (*P* < 0.05). The ileal mucosa *SLC7A2* expression in the T1 treatment was lower than that in the T0 treatment (*P* < 0.05). There was no interaction between dietary treatment and days post-weaning on amino acid transferase expression. In addition, we tested the Na^+^-K^+^ ATPase activities in jejunal and ileal mucosa (Table 8). Compared with day 5 post-weaning, the Na^+^-K^+^ ATPase activity was significantly increased on day 21 post-weaning (*P* < 0.05). Days post-weaning did not affect the diet treatments' efficacy of the serum of amino acid transporter gene expression and Na+-K+- ATPase activity in the small intestine of weaning piglets (*P* > 0.05).

**Table 7:** Relative gene expressions of amino acid transporters in the ileal and jejunal mucosa of weaning piglets.

**Item**	**Diet treatment**			**Day post-weaning**		**SEM**			***P*** **-value**	
	**T0**	**T1**	**T2**	**5**	**21**	**Diet**	**Day**	**Diet**	**Day**	**Diet**×**Day**
**Jejunal mucosa**										
*SLC1A1*	1	0.56	0.83	1.03	0.56	0.209	0.17	0.331	0.058	0.955
*SLC1A5*	1	0.4	1.34	1.21	0.62	0.346	0.282	0.166	0.155	0.241
*SLC3A1*	1	0.68	0.76	0.88	0.75	0.211	0.172	0.545	0.581	0.208
*SLC3A2*	1	0.55	0.73	0.89	0.63	0.145	0.118	0.105	0.123	0.413
*SLC36A1*	1	0.88	0.97	1.05	0.85	0.195	0.162	0.912	0.406	0.075
*SLC7A2*	1.00^ab^	1.38^a^	0.07^b^	0.89	0.74	0.337	0.275	0.029	0.707	0.291
*SLC38A2*	1	0.44	2.01	0.7	1.61	0.595	0.486	0.184	0.193	0.635
*SLC6A19*	1	1.09	0.62	1.12	0.68	0.186	0.152	0.181	0.053	0.604
*SLC6A20*	1	1.34	0.7	1.31^*^	0.71	0.226	0.185	0.154	0.029	0.634
*SLC7A1*	1	1.14	0.64	1.19^*^	0.66	0.179	0.146	0.14	0.014	0.754
*SLC7A7*	1	0.57	1.18	1.49^*^	0.34	0.279	0.227	0.288	0.001	0.208
*SLC7A9*	1	0.83	0.79	1.23^*^	0.51	0.196	0.16	0.721	0.003	0.977
*SLC16A10*	1	0.91	0.96	1.47^*^	0.44	0.194	0.159	0.942	0	0.994
*SLC38A9*	1	0.92	0.79	1.68^*^	0.13	0.262	0.214	0.85	0	0.936
*SLC6A14*	1	1.43	0.44	1.03	0.89	0.574	0.468	0.482	0.844	0.357
*SLC7A11*	1	0.13	0.95	0.11	0.08	0.023	0.019	0.057	0.265	0.845
**Ileal mucosa**										
*SLC1A1*	1	0.26	0.51	0.23	0.95^*^	0.228	0.186	0.083	0.01	0.077
*SLC1A5*	1.00^ab^	0.34^b^	1.29^a^	0.99	0.76	0.265	0.216	0.047	0.439	0.761
*SLC3A1*	1.00^ab^	0.34^b^	1.45^a^	0.66	1.20^*^	0.196	0.16	0.002	0.021	0.23
*SLC3A2*	1.00^b^	0.37^c^	1.63^a^	1.12	0.88	0.151	0.124	0	0.19	0.163
*SLC36A1*	1.00^b^	0.79^b^	1.68^a^	1.39^*^	0.92	0.179	0.146	0.004	0.029	0.873
*SLC7A2*	1.00^a^	0.11^b^	0.61^ab^	0.54	0.61	0.193	0.158	0.01	0.731	0.902
*SLC38A2*	1	1.56	1.99	1.9	1.13	0.337	0.275	0.134	0.056	0.517
*SLC6A19*	1	0.78	0.86	0.86	0.9	0.163	0.133	0.63	0.858	0.445
*SLC6A20*	1	1.08	1.15	0.93	1.22	0.132	0.108	0.72	0.064	0.704
*SLC7A1*	1	1.18	1.21	1.38^*^	0.88	0.115	0.094	0.391	0.001	0.459
*SLC7A7*	1	1.33	1.4	0.91	1.57^*^	0.158	0.129	0.181	0.001	0.997
*SLC7A9*	1	1.15	1.2	1.1	1.13	0.143	0.117	0.592	0.878	0.973
*SLC16A10*	1	1.23	1.32	1.07	1.3	0.179	0.146	0.435	0.277	0.953
*SLC38A9*	0.99	0.96	1.04	1.17^*^	0.82	0.083	0.068	0.802	0.001	0.269
*SLC6A14*	1.00^b^	0.67^b^	1.78^a^	1.29	1	0.213	0.174	0.003	0.247	0.278
*SLC7A11*	1.00^b^	1.08^b^	2.58^a^	1.81	1.3	0.445	0.363	0.029	0.325	0.561

^a, b^Values with different letters within the same row are different (*P* < 0.05); ^*^means the difference was significant when compared to d 5 post-weaning (*P* < 0.05); *n* = 6. The results are presented as main effects, and the differences among treatments or days are evaluated using Tukey's HSD test.

**Table 8:** Na^+^-K^+^- ATPase activities in the small intestinal mucosa of weaning piglets.

**Item**	**Diet treatment**			**Day post-weaning**		**SEM**			***P*** **-value**	
	**T0**	**T1**	**T2**	**5**	**21**	**Diet**	**Day**	**Diet**	**Day**	**Diet**×**Day**
Jejunal mucosa	2.38	2.6	2.8	2.11	3.18^*^	0.155	0.219	0.103	0	0.081
Ileal mucosa	2.02	2.79	2.57	2.06	2.85^*^	0.242	0.342	0.091	0.008	0.205

^*^means the difference was significant when compared to d 5 post-weaning (*P* < 0.05); *n* = 6. The results are presented as main effects, and the differences among treatments or days are evaluated using Tukey's HSD test.

### 3.5. Asp, glu, and gln stimulated the mtor signaling pathway of jejunal and ileal mucosa in weaning piglets

**Figure 4:**
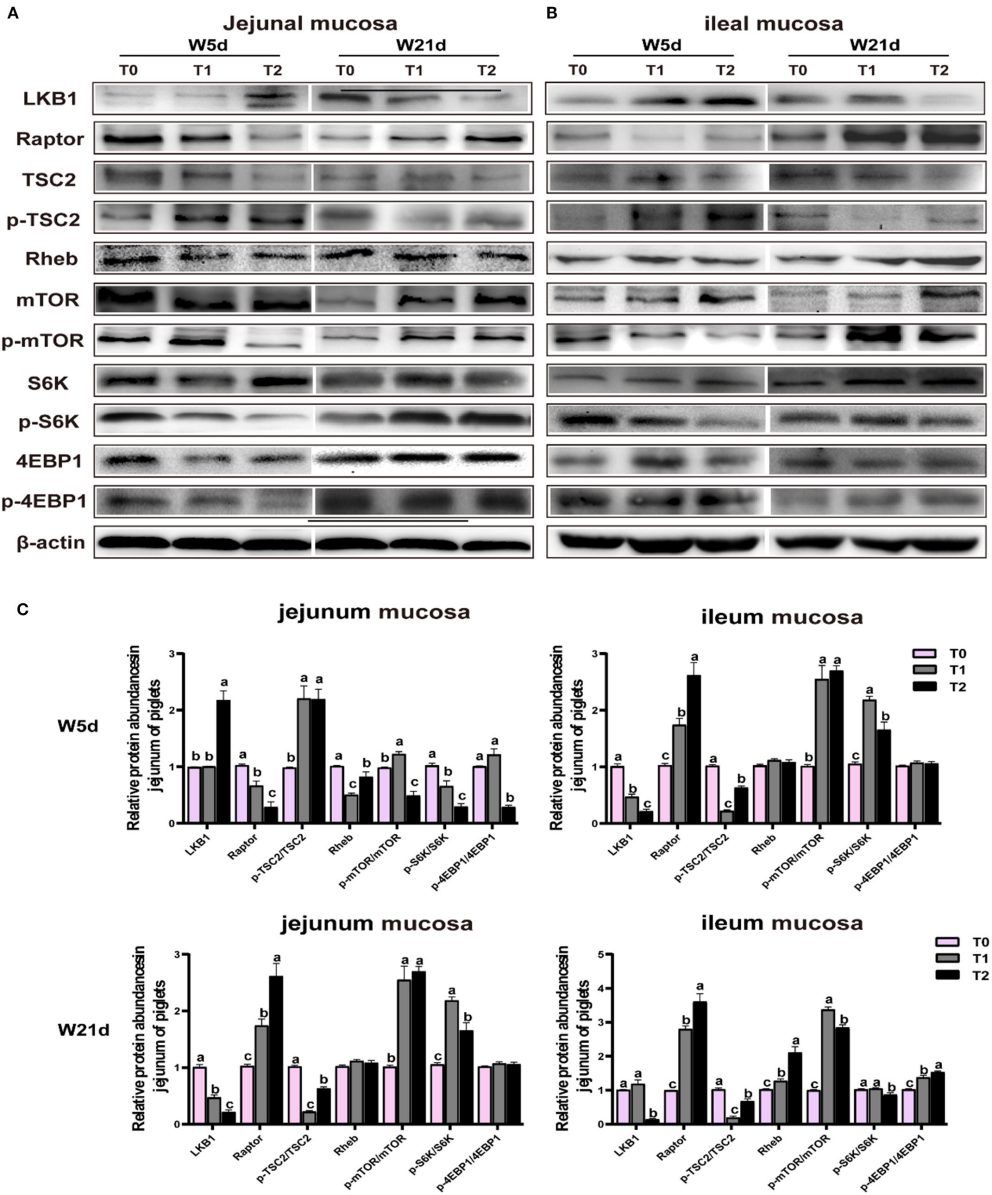
Relative protein abundances of mTOR signaling pathway in **(A)** and ileal **(B)** mucosa of weaning-piglets. **(C)** mTOR signaling pathway of densitometric quantification. Data are presented as the simple main effect analysis and expressed as means ± SEM, *n* = 6. ^a − c^Values with different lowercase letters are 506 different (*P* < 0.05).

The relative protein abundances of the mTOR pathway in jejunal and ileal mucosa are presented in Table 9 and Figure 4. In the jejunal mucosa, T1 treatment significantly increased the ratio of p-mTOR to mTOR and the ratio of p-4EBP1 to 4EBP but decreased the LKB1 protein abundance as compared with the T0 and T2 treatments (*P* < 0.05). In comparison to the T1 treatments, the T2 treatment decreased the ratio of p-S6K to S6K (*P* < 0.05). In the ileal mucosa, T2 treatment enhanced the relative protein abundances of Raptor, Rheb, p-TSC2/TSC2, and p-4EBP1/4EBP but declined the ratio of p-mTOR to mTOR, and the ratio of p-S6K to S6K as compared with the T1 treatment (*P* < 0.05). In comparison to the T0 treatment, the T1 treatment increased the protein expression levels of LKB1 and Raptor and the ratio of p-mTOR to mTOR (*P* < 0.05).

**Table 9:** Relative protein abundances of the mTOR signaling pathway of jejunal and ileal mucosa of weaning piglets.

**Item**	**Diet treatment**			**Day post-weaning**		**SEM**			***P*** **-value**	
	**T0**	**T1**	**T2**	**5**	**21**	**Diet**	**Day**	**Diet**	**Day**	**Diet**×**Day**
**Jejunal mucosa**										
LKB1	1.00^a^	0.07^b	1.20^a^	1.39^*^	0.57	0.052	0.043	0	0	0
Raptor	1.03^b^	1.21^ab	1.45^a^	0.66	1.80^*^	0.081	0.067	0.011	0	0
p-TSC2/TSC2	1.01^b^	1.22^ab	1.42^a^	1.80^*^	0.63	0.082	0.067	0.014	0	0
Rheb	1.02^a^	0.81^b	0.95^ab^	0.78	1.08^*^	0.031	0.025	0.002	0	0
p-mTOR/mTOR	1.00^c^	1.89^a	1.60^b^	0.9	2.09^*^	0.078	0.063	0	0	0
p-S6K/S6K	1.04^a^	1.42^a	0.98^b^	0.66	1.66^*^	0.055	0.045	0	0	0
p-4EBP1/4EBP	1.02^b^	1.15^a	0.68^c^	0.84	1.05^*^	0.034	0.028	0	0	0
**Ileal mucosa**										
LKB1	1.01^b^	1.38^a	1.34^a^	1.71^*^	0.078	0.072	0.058	0.005	0	0
Raptor	1.01^c^	1.67^b	2.13^a^	0.74	2.468^*^	0.079	0.065	0	0	0
p-TSC2/TSC2	1.02^b^	0.95^b	1.51^a^	1.68^*^	0.631	0.048	0.039	0	0	0
Rheb	1.02^b^	1.26^b	1.59^a^	1.11	1.470^*^	0.074	0.06	0.001	0.001	0
p-mTOR/mTOR	1.00^c^	1.97^a	1.58^b^	0.62	2.406^*^	0.036	0.029	0	0	0
p-S6K/S6K	1.03^a^	0.92^a	0.58^b^	0.69	0.981^*^	0.033	0.027	0	0	0
p-4EBP1/4EBP	1.01^b^	0.96^b	1.19^a^	0.8	1.311^*^	0.024	0.019	0	0	0

^a − c^Values with different letters within the same row are different (*P* < 0.05); ^*^means the difference was significant when compared to d 5 post-weaning (*P* < 0.05); *n* = 6. The results are presented as main effects, and the differences among treatments or days are evaluated using Tukey's HSD test.

Meanwhile, days post-weaning affect the efficacy of diet treatments on the mTOR pathway stimulation (*P* < 0.05). As shown in Figure 4, on day 5 post-weaning as compared with T0 treatment, T1 treatment significantly decreased the jejunal and ileal mucosa Raptor and jejunal mucosa LKB1 protein abundances, as well as jejunal mucosa p-S6K/S6K, and ileal mucosa p-mTOR/mTOR, p-S6K to S6K, and p-4EBP1/4EBP1 (*P* < 0.05) while increased jejunal mucosa p-TSC2/TSC2 and p-mTOR/mTOR and ileal mucosa LKB1 protein abundance and p-mTOR/mTOR (*P* < 0.05). In comparison to the T1 treatment, the T2 treatment reduced the Raptor protein abundance, p-mTOR/mTOR, p-S6K/S6K, and p-4EBP1/4EBP1 in the jejunal mucosa, and p-mTOR/mTOR and p-S6K/S6K in the ileal mucosa (*P* < 0.05), but increased the LKB1 and Rheb protein abundances in the jejunal mucosa and LKB1 protein abundance, and p-TSC2/TSC2 and p-4EBP1/4EBP1 in the ileal mucosa (*P* < 0.05). On day 21 post-weaning, as compared with T0 treatment, T1 treatment decreased the jejunal mucosa LKB1 protein abundance and jejunal and ileal mucosa p-TSC2/TSC2 (*P* < 0.05) while increasing the jejunal mucosa Raptor, p-mTOR/mTOR, and p-S6K/S6K, ileal mucosa Raptor and Rheb protein abundances, and the ileal mucosa p-mTOR/mTOR and p-4EBP1/4EBP1 (*P* < 0.05). In comparison to the T1 treatment, T2 treatment enhanced the jejunal and ileal mucosa Raptor and p-TSC2/TSC2 and ileal mucosa Rheb protein abundance and p-4EBP1/4EBP1 (*P* < 0.05) whereas decreased the jejunal and ileal mucosa LKB1 protein abundance and p-S6K/S6K, as well as the ileal mucosa p-mTOR/mTOR (*P* < 0.05).

## 4. Discussion

Weaning stress leads to lower nutrient absorption capacity and lower energy intake, resulting in significant body weight loss and affecting production performance (18, 19). Recently, numerous studies have shown the positive effects of gln, glu, and asp as nutritional additives for piglets (10, 20, 21). Our previous studies have shown that gln, glu, and asp could improve small intestinal energy homeostasis and affect hepatic lipid metabolism in post-weaning piglets (22, 23). The current study aimed to investigate the regulation of gln, glu, and asp on the amino acid pool, intestinal barrier integrity, and protein synthesis with different energy level diets in weaned piglets.

Premature weaning commonly affects intestinal barrier integrity, resulting in gastrointestinal disorders, inflammation, and diarrhea in piglets (24). In the present study, supplementation with gln, glu, and asp in a normal energy diet could increase the expressions of the tight junction proteins occludin, claudin-1, and claudin-3 in the small intestine, while the beneficial effect in a low energy diet was slightly inferior. The serum concentrations of D-lactate and DAO could reflect the degree of intestinal mucosal damage, and their elevated levels indicate an increase in the intestinal permeability (25). Dietary supplementation with gln, glu, and asp decreased serum DAO and D-lactate levels in weaning piglets. In addition, supplementation of gln, glu, and asp increased the positive rate of PCNA in the jejunum. These results may suggest that dietary supplementation with gln, glu, and asp improved the intestinal barrier integrity by promoting intestinal epithelial cell proliferation and maintaining the structure of tight junction.

Amino acids are essential for intestinal growth and barrier function (26). Dietary supplementation with gln, glu, and asp changed the amino acid pool in the serum of piglets. The treatments of gln, glu, and asp under different energy levels have shown different effects on the amino acid pool. Gln, glu, and asp supplementation increased serum asp levels in a normal energy diet while raising serum phe, lys, arg, and thr levels in a low-energy diet. Generally, exogenous supplementation of a certain amino acid might affect its concentration and metabolism in hosts, such as dietary asp or trp addition elevates the serum asp and trp levels and changes the trp metabolite contents (27, 28). However, supplementation of gln, glu, and asp in our trials did not increase serum glu and asp levels under the low-energy diets. This result may be explained by the low energy intake of piglets, which makes amino acids are used to replenish Krebs cycle or convert to other amino acids or bioactive molecules, including glutamine, glutathione, arginine, purines, and pyrimidines. Glu can be formed into a-ketoglutarate and alanine by the action of pyruvate aminotransferase, which enters the tricarboxylic acid cycle to produce energy (10, 29). The amino acid anabolism, metabolism, and interconversion changed continuously within 1 week after weaning (11). In the present study, the effects of gln, glu, and asp on the serum amino acid profile were not consistent, which may be explained by the utilization of specific amino acids at different stages after weaning. The most severe stage of weaning-stress damage is 3–5 days after weaning, and the piglets have recovered by day 21 post-weaning.

The absorption and transportation of lumen amino acids depend on their specific transporter system including the ion channels on the apical side and the ion pumps on the basal layer (30). In our study, gln, glu, and asp treatment mainly stimulated the expression of amino acid transporters of piglets fed with a low-energy diet. The *slc1a5, slc3a1, slc3a2, slc7a11, slc36a1*, and *slc6a14* mRNA expressions were raised by gln, glu, and asp treatment. The *slc1a5, slc3a1, slc3a2*, and *slc7a11* are highly specific to the extracellular uptake of cys and release of glu so as to promote glutathione synthesis and protect cells from oxidative damage (31, 32). The *slc36a1* is an absorptive intestinal transporter for α-amino acids, such as pro, gly, and ala (33). In addition, Na^+^-K^+^ ATPase locates in the lateral basement membrane of intestinal epithelial cells and is instrumental in much of amino acid uptake by transferring K^+^ ions into and Na^+^ out of the cell (34). We found that the Na^+^-K^+^ ATPase activity on day 5 post-weaning was lower than that on day 21 post-weaning. It is possible that the day 5 post-weaning leads to severe intestinal damage, and the requirement for amino acids is higher in the damage repair process, while Na^+^-K^+^ ATPase can accelerate the transport of amino acids in the intestinal cavity during this process (30). The changes in amino acid transferases and ion pumps in the small intestine indicated that gln, glu, and asp treatment might promote amino acid absorption under a low-energy diet, which is consistent with the change in serum amino acid profile.

LKB1 is known to be an Arg/Asn homeostasis sensor and a regulator of cell metabolism and growth (35). LKB1 is a critical kinase that phosphorylates and activates AMPK, which further phosphorylates the downstream raptor and TSC2. The activation of TSC2 leads to the inhibition of mTOR signaling via inactivation of the small GTPase RheB (36). It has been shown that mTOR plays a key role in synthesizing cellular protein synthesis and controlling cell growth by recruiting downstream substrates such as eukaryotic initiating factors 4EBP1 and S6K. An *in vitro* study using intestinal porcine epithelial cells (IPEC-1) showed that gln supplementation was able to promote enterocyte growth by activating the mTOR-4EBP1 signaling pathway (37). Consistently, we found that supplementation with gln, glu, and asp could activate raptor and phosphorylate mTOR and its downstream effective factor S6K and 4EBP1 proteins in the small intestine of piglets on day 21 post-weaning. However, on day 5 post-weaning, the phosphorylation levels of mTOR signaling in the small intestine of piglets with gln, glu, and asp administration were lower than those in the basal diet treatments. The different efficacy of the intervention on the LKB1-TCS2-mTOR signaling pathway affected by different stages after weaning (day 5 or day 21 post-weaning) was consistent with our previous study (11). Our previous study has shown the gln, glu, and asp treatment differentially regulates the phosphorylation of the AMPK signaling pathway (11). Here, we speculated that the different activation of mTOR signaling might be due to glu, gln, and asp mainly used to restore the intestinal energy homeostasis by replenishing the Krebs cycle but not used in protein synthesis on day 5 post-weaning, especially under the low energy diet. While on day 21 post-weaning, the injury of intestinal mucosa has been repaired, and the barrier function tends to be more mature, which makes the enterocyte more inclined to use amino acids for protein synthesis so as to promote the rapid growth of the host.

## 5. Conclusion

In conclusion, we have found that supplementation of gln, glu, and asp in a normal or low energy level diet could improve small intestinal barrier integrity and regulate the amino acid pool to promote the phosphorylation of the mTOR signaling pathway. Moreover, the development and maturity of the intestine of piglets might affect the efficiency of gln, glu, and asp treatment, which implied that the optimal window-period and the duration of treatment should be considered in the formulation of nutrition intervention strategies.

## Data availability statement

The raw data supporting the conclusions of this article will be made available by the authors, without undue reservation.

## Ethics statement

The animal study was reviewed and approved by the Hunan Agricultural University. Written informed consent was obtained from the owners for the participation of their animals in this study.

## Author contributions

YD, BT, JW, JiL, and HC contributed to conception and design of the study. JuL, HH, MQ, and NW organized the database. YD and HC performed the statistical analysis. YD wrote the first draft of the manuscript. YD, HC, JiL, and JW wrote sections of the manuscript. All authors contributed to manuscript revision, read, and approved the submitted version.
